# Health Tract, No. 230

**Published:** 1865

**Authors:** 


LIKE pain, is the faithful sentinel giving timely warning of approaching danger; it never raises a false alarm; it never comes without an adequate cause, and if it were promptly heeded and properly treated, myriads of lives would be saved, and an incalculable amount of sickness and suffering would be prevented every year. Cough ought always to be encouraged, and yet an impression prevails almost universal that whatever "helps," that is to say, whatever lessens the cough, "does good," when in reality it is positively, directly, and always injurious, for the following reason, which will carry with it a resistless conviction, in every mind of even moderate reflecting powers. All know that when a crumb of bread, ever so small, "goes the wrong way," that is, passes down the wind-pipe into the lungs, there is an irrepressible tickling sensation felt in the throat, inducing a dry, spiteful, and persistent cough, which is continued until the offending intruder is cast out; this crumb was in no part a portion of the lungs, nor was necessary to their healthful action; it was a foreign body, and as such, was offensive, and its presence could not be tolerated; this is a universal law of our being, that when any thing is in the body, which is not a part of it, nature, in one way or another, sets about ejecting it, and will keep at it for days and weeks, and months even, until the object is accomplished; as proof, the issue of needles, pins, and other sharp substances at some inches and even feet from the spot where they entered. As to more blunt articles, bullets and the like, more or less discomfort is at first experienced; but when they can not be removed, a kind of instinct seems to ascertain the fact, when a new process is set on foot, a gristly substance is thrown out and grows around the foreign body, incloses it in a prison, where it may remain innocuous, comparatively, for life. The lungs, in health, are always throwing out, manufacturing, a thin, mucilaginous-like liquid, near the color of the white of an egg, for the purpose of lubrication, so that they rise and fall at each breath with facility, without friction. This "mucus" is a part of the lungs, a part of their healthful product, and its presence causes no disturbance; but a common cold falling on the lungs changes the color and consistence of this lubricating material, and it becomes yellow and thick; and this being unnatural, becomes at once a foreign body; nature grows uneasy and sets up a cough to aid in its ejection, as if it were a crumb of bread which had gone the wrong way. When a cough begins to dislodge this, it comes up in the shape of yellow matter; the cold is said to "break," and the person begins to get well. Whatever represses cough, as all cough-drops, lozenges, troches do, only keeps this yellow matter longer in the lungs; only protracts the cure; but if kept in too long, nature makes the attempt to get rid of it in another way, by reäbsorbing the yellow matter and throwing it into the general circulation again; evidenced by a red spot in one or both cheeks, called "Hectic," at the same time night-sweats come on, and this is consumption in its last stages! When will people become wise?

